# Versatile Aggressive Mimicry of Cicadas by an Australian Predatory Katydid

**DOI:** 10.1371/journal.pone.0004185

**Published:** 2009-01-14

**Authors:** David C. Marshall, Kathy B. R. Hill

**Affiliations:** Department of Ecology and Evolutionary Biology, University of Connecticut, Storrs, Connecticut, United States of America; Queens University, Canada

## Abstract

**Background:**

In aggressive mimicry, a predator or parasite imitates a signal of another species in order to exploit the recipient of the signal. Some of the most remarkable examples of aggressive mimicry involve exploitation of a complex signal-response system by an unrelated predator species.

**Methodology/Principal Findings:**

We have found that predatory *Chlorobalius leucoviridis* katydids (Orthoptera: Tettigoniidae) can attract male cicadas (Hemiptera: Cicadidae) by imitating the species-specific wing-flick replies of sexually receptive female cicadas. This aggressive mimicry is accomplished both acoustically, with tegminal clicks, and visually, with synchronized body jerks. Remarkably, the katydids respond effectively to a variety of complex, species-specific Cicadettini songs, including songs of many cicada species that the predator has never encountered.

**Conclusions/Significance:**

We propose that the versatility of aggressive mimicry in *C. leucoviridis* is accomplished by exploiting general design elements common to the songs of many acoustically signaling insects that use duets in pair-formation. Consideration of the mechanism of versatile mimicry in *C. leucoviridis* may illuminate processes driving the evolution of insect acoustic signals, which play a central role in reproductive isolation of populations and the formation of species.

## Introduction

In aggressive mimicry, a predator or parasite imitates a signal of another species in order to exploit the recipient of the signal. In some of the most remarkable cases, a predator species mimics complex sexual signals of its prey. The bolas spider (*Mastophora* sp.) attracts male moths of at least two species with a chemical imitation of moth sex pheromones [1], [2]. The predaceous firefly *Photuris versicolor* lures male *Photinus* fireflies by mimicking female reply flashes, which have a specific timing in relation to the male's signal [3], [4]. *Photuris versicolor*'s mimicry is especially striking because of its versatility – the predator is able to mimic the species-specific female replies of up to eleven different prey species. Developing plausible adaptive hypotheses to account for the evolution of such complex mimicry is an intriguing challenge. In the case of *Photuris* and *Photinus*, at least, the close phylogenetic relatedness of predator and prey is likely involved.

In this paper we present a striking example of aggressive mimicry involving taxonomically unrelated predator and prey and an unusual degree of versatility. The Spotted Predatory Katydid, *Chlorobalius leucoviridis* (Orthoptera: Tettigoniidae), lures male cicadas of the Tribe Cicadettini (Hemiptera: Cicadidae) by imitating species-specific, acoustic reply signals of female cicadas. This remarkable predator is able to mimic a large number of species, including those with which it has never interacted historically. In this case, aggressive mimicry appears to have been facilitated by a design constraint common to certain acoustic duetting communication systems, including that of Cicadettini cicadas and some katydids.

**Figure 1:**
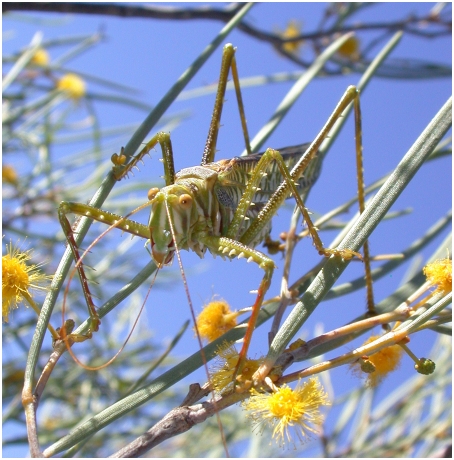
Male *Chlorobalius leucoviridis* Spotted Predatory Katydid.

**Figure 2:**
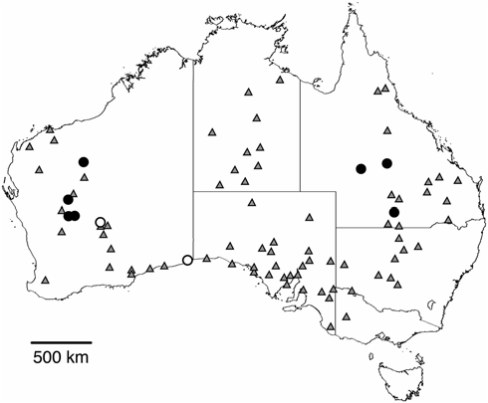
Map of the known distribution of *Chlorobalius leucoviridis* modified from Rentz [41] (grey triangles). Solid black dots show locations where katydids were collected for this study. White dots represent locations where *C. leucoviridis* were heard making their calling song and/or seen by the authors but not collected.

**Figure 3:**
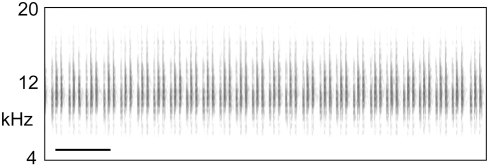
Calling song of a *Chlorobalius leucoviridis* male from Cunnamulla, QLD. The scale bar in the lower left represents 500 ms.

*C. leucoviridis* is a large, cryptically colored, green-and-white katydid of the subfamily Listroscelidinae [5] (Fig. 1). It is found throughout the arid interior of Australia (Fig. 2). Adults are active in the summer and can be found in the tops of large shrubs and small trees during both daytime and nighttime. Both males and females possess file-and-scraper structures on the forewings (tegmina), and males make loud irregularly broken trilling songs (Fig. 3) at night to attract conspecific females. Female acoustic behavior is not yet known, but it is likely that female *C. leucoviridis* silently approach a calling male and mate (see Discussion).

**Figure 4:**
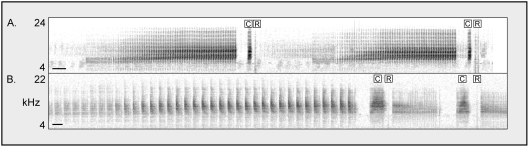
Sonograms of field-recorded male-female duets from two Cicadettini species: (A) *Maoricicada campbelli*; (B) *Kikihia* sp. “flemingi”. Each male song cue is marked with a “C”, and each female wing-flick response is marked with an “R”. The scale bar in the lower left of each diagram represents 100 ms. In A, faint background songs of conspecifics and crickets are visible.

The cicada tribe Cicadettini contains hundreds of Australian species, most of them undescribed [6],[7 and unpublished data]. Species of this group dominate the cicada fauna of interior Australia where *C. leucoviridis* is found [unpublished data]. Most cicadettine species employ stereotyped signal-response “duets” [8] during sexual pair-formation [9],[10] ,[11 p. 211],[12 p. 1056, and K. B. R. Hill and D. C. Marshall unpublished data]. Males sing a species-specific calling song containing a particular song element or echeme that triggers “wing-flick” responses from nearby sexually receptive females [for acoustic terminology see 13]. The female responses are simple, brief (1 ms), broad-frequency sounds, and they are sometimes audible from many meters away. Because a wing-flick reply is structurally nondescript, it must closely follow the cue in the male cicada's song in order to be recognized (Fig. 4); we have measured reply latencies of 29–68 ms across a limited number of species [K. B. R. Hill and D. C. Marshall unpublished data]. Males locate females by listening for these responses and by searching visually for wing-flicking females when at close range [cf. 14]. We have found that males of many Cicadettini can be attracted by sounds like finger-snaps if they follow the correct song echeme within roughly 100 ms, although differences between species in wariness and strictness of reply timing make some species more difficult to attract than others. This technique has allowed us to identify the correct position of the female reply for hundreds of cicada species.

## Results

### Discovery of aggressive mimicry in *Chlorobalius leucoviridis*

At a location near Cunnamulla, Queensland, in 2005, we noticed on two occasions what sounded like loud female wing-flick replies to male *Kobonga oxleyi* (Distant) cicadas. In the first case, the male cicada approached to within 30 cm of the responder but then suddenly flew away. The second cicada male was audio-recorded while singing from ca. 10 m away from two different responders. We soon discovered that the replies were being made not by female cicadas, but by predatory *Chlorobalius leucoviridis* katydids. Direct observations of clicking male katydids later confirmed that the wings move with each click, so the sound is probably made using the stridulatory apparatus.

**Figure 5:**
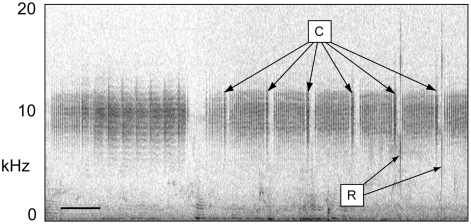
Segment of a field recording showing clicks from a wild *Chlorobalius leucoviridis* placed after two of the male song cues made by a wild *Kobonga oxleyi*. A segment of this recording is available online in the supplementary material (Audio S1). The scale bar in the lower left represents 1 s.

A sonogram of the field interaction from Cunnamulla (Fig. 5) shows that the katydid clicks closely resemble cicada female wing-flicks in sound content and timing (compare with Fig. 4). The delay between the cicada song cue and the katydid click reply averages 58 ms at 31.6°C air temperature (n = 10 replies, SD = 3 ms), within the range of reply latencies observed in cicadettine cicadas. Most importantly, each of the *C. leucoviridis* clicks follows one of the *K. oxleyi* song cues (108 replies in two minutes).

We began searching for *Chlorobalius leucoviridis* katydids during our cicada-collecting trips to obtain specimens for observations, playbacks, and predation trials. In all, we collected 12 male and 2 female *C. leucoviridis* from seven sites (Table 1) during 2005–2008. Four males were collected responding to cicadas (three to *K. oxleyi*, one to an undescribed *Kobonga*). The other eight males were collected after tracking their calling songs at night (Fig. 3), and the two females were found fortuitously in vegetation. On three occasions we heard what sounded like *C. leucoviridis* clicking back to cicadas but we could not locate the sound source.

**Table 1:** Collecting localities for *Chlorobalius leucoviridis* specimens used in this study (see also Fig. 2).

Number Collected	Date	Lat.	Lon.	Location
3*	4-Jan-05	−27.71	145.90	ca. 44 km NNE of Cunnamulla, QLD
4	28-Jan-06	−27.71	145.90	ca. 44 km NNE of Cunnamulla, QLD
2	12-Feb-06	−23.52	119.77	3 km S. of Capricorn Roadhouse, WA
1 (female)	14-Feb-06	−28.01	119.00	30.5 km W of Sandstone, WA
1 (female)	16-Feb-06	−28.03	118.53	Rest area 77 km W of Sandstone, WA
1	17-Feb-06	−28.58	121.20	36.5 km N of Leonora, WA
1*	7-Feb-08	−23.65	145.28	9.7 km S of Barcaldine, QLD
1	12-Feb-08	−24.09	143.14	Noonbah Stn., SW of Longreach, QLD

* indicates specimens collected because they were heard responding to cicadas in the wild.

### Demonstrations of versatile aggressive mimicry

*Chlorobalius leucoviridis* katydids demonstrated remarkable versatility in their response to cicadettine cicada songs. In approximately 30 minutes of accumulated digital recordings of field observations, playback trials, and recordings of caged cicadas interacting with caged *C. leucoviridis*, the katydids clicked after the correct song echemes more often than not for 22 out of 26 species (Table 2). In 18 cases, the katydids responded to the correct echemes more than 90% of the time. Furthermore, in 9 out of the 10 cases in which the cicada species' song structure allowed a straightforward classification of echemes into “cueing” and “noncueing” elements (see Methods), the association of katydid replies with cueing echemes was statistically significant (Table 2). Observations were made of *C. leucoviridis* clicking in response to four additional species that were not tape-recorded. Both male and female katydids were observed responding to cicadas, although most observations were made with males.

**Table 2:** Accuracy of *Chlorobalius leucoviridis* click replies in response to songs of different Cicadettini species.

Obs.	Genus	Species	# Echemes	# Cues	# Replies	# Correct	*P*<
Cage	*Cicadetta*	*hackeri*	-	-	11	11	-
Cage	*Cicadetta*	viridis*	-	-	23	23	-
Cage	*Kobonga*	*apicans*	96	42	40	24	0.028
Cage	*Pauropsalta*	*melanopygia*	36	33	20	19	0.497
Cage	*Pauropsalta*	“Sandstone”*	22	16	17	16	0.034
Cage	*Urabunana*	marshalli*	-	-	37	36	-
Cage	N. Gen.	“pale grass cicada”*	-	-	117	80	-
Cage	N. Gen.	“near pale grass cicada”	-	-	29	25	-
Cage	N. Gen.	“swinging tigris”*	218	60	68	32	0.001
Cage	N. Gen.	“Kynuna”*	-	-	48	48	-
Cage	N. Gen.	“revving tigris”	-	-	43	8	-
Cage	N. Gen.	“northwestern”	-	-	40	37	-
Cage	N. Gen.	“troublesome tigris”*	-	-	47	8	-
Cage	N. Gen.	“Nullarbor wingbanger”*	-	-	31	28	-
Playback	*Cicadetta*	calliope*	-	-	3	3	-
playback	*Kikihia*	*angusta*	-	-	36	34	-
playback	*Kikihia*	*cauta*	-	-	4	4	-
playback	*Kikihia*	“aotea”	340	310	152	150	0.000
playback	*Kikihia*	“nelsonensis”*	500	253	20	18	0.000
playback	*Kikihia*	*rosea*	-	-	10	10	-
playback	*Kikihia*	scutellaris*	-	-	25	1	-
playback	*Kikihia*	subalpina*	32	16	11	11	0.000
playback	*Kikihia*	“tuta”*	75	37	36	35	0.000
playback	*Maoricicada*	campbelli*	68	34	43	34	0.000
playback	*Maoricicada*	*mangu*	32	16	6	6	0.016
Field	*Kobonga*	oxleyi*	-	-	108	108	-
Cage	*Pauropsalta*	“near walkeri”				Many	
Cage	*Pauropsalta*	“near extrema”				Many	
Field	*Pauropsalta*	*annulata*				Many	
Field	N. Gen.	“tigris H2”				Many	
Field	*Kobonga*	*umbrimargo*				None	

Obs: Observation type (caged cicadas and katydids, playback of cicada song to caged katydids, or field observations). # Echemes: Number of song echemes in recording. # Cues: Number of echemes that cue female replies. # Replies: Number of katydid clicks in recording. # Correct: Number of katydid replies that follow a song cue. Tallies are not available for five anecdotal field observations; however in all cases except *K. umbrimargo*, for which only a brief interaction was observed, many katydid clicks followed song cues. * indicates cicada songs included in Figs. 5–7. Temporary field nicknames for undescribed taxa are indicated by quotation marks. *P*-value shows a binomial test of association of katydid replies with cicada song cues (see Methods).

**Figure 6 pone-0004185-g006:**
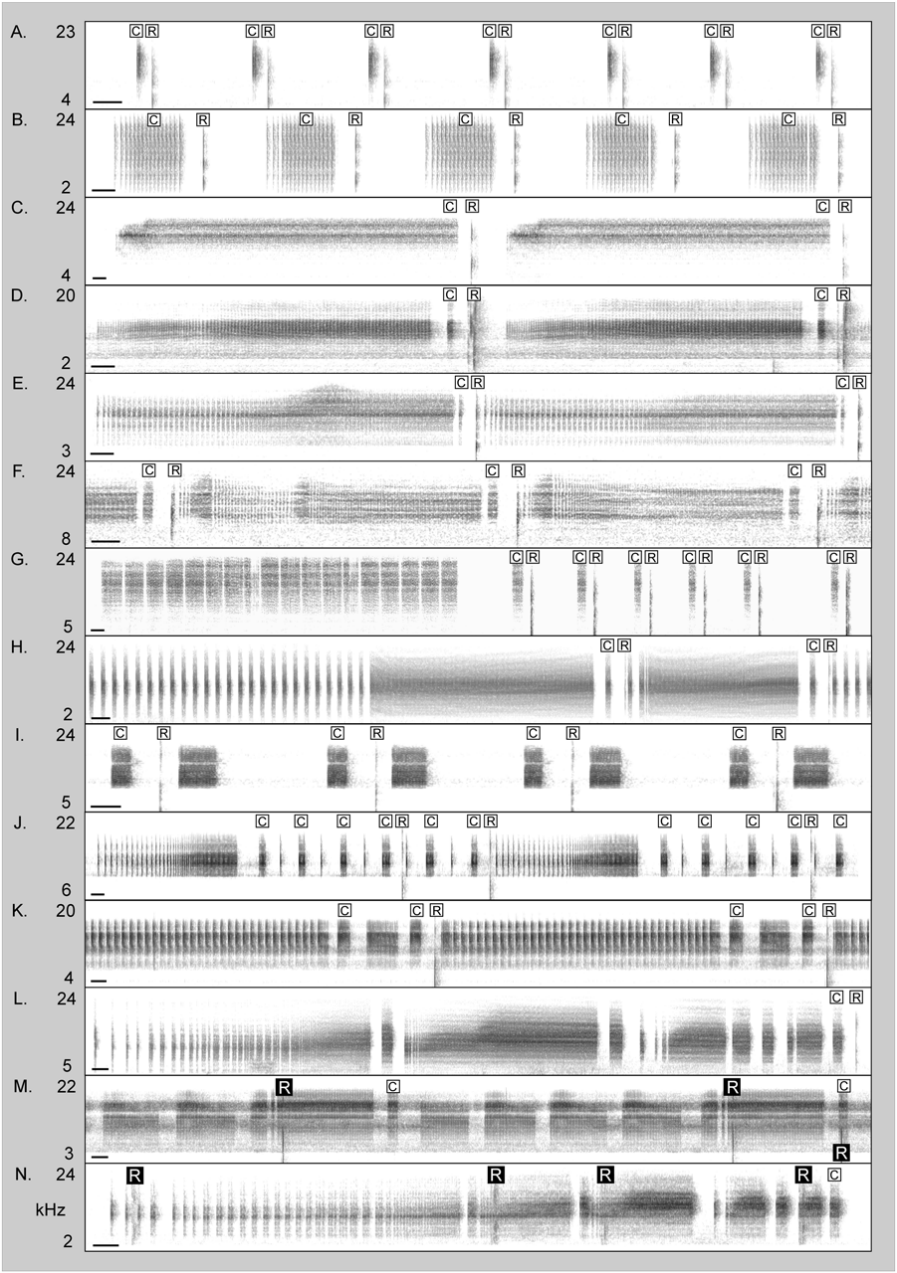
Demonstrations of versatile aggressive mimicry in a predatory katydid. Sonograms show *Chlorobalius leucoviridis* click replies (marked with “R”) produced in response to cues (marked with “C”) of songs of 14 Cicadettini species from at least nine genera: (A) *Urabunana marshalli* – Australia (AUS) ; (B) Undesc. genus, sp. “Nullarbor wingbanger” – AUS; (C) *Cicadetta calliope* – USA; (D) *Maoricicada campbelli* – New Zealand (NZ); (E) Undesc. genus, sp. “Kynuna” – AUS; (F) Undesc. genus., sp. “pale grass cicada” – AUS; (G) *Cicadetta viridis* – AUS; (H) *Pauropsalta* sp. “Sandstone” – AUS; (I) *Kikihia* sp. “tuta” – NZ; (J) *Kikihia* sp. “nelsonensis”; (K) *Kikihia subalpina* – NZ; (L) Undesc. genus, sp. “swinging tigris” – AUS; (M) *Kikihia scutellaris* – NZ; (N) Undesc. genus, sp. “troublesome tigris” – AUS. A white ‘R’ in a black box (in M and N) indicates an incorrect reply, all other katydid replies are correctly placed. The katydid responses in C, D, I, J, K, and M were made to playbacks of recorded and filtered cicada songs; the remainder of the illustrations show live recordings of katydids replying to cicadas in cages. In D, two katydids are responding. Audio recording of the interaction in H is available online in Supplementary Material (Audio S2). The scale bar in the lower left of each diagram represents 100 ms.

Cicada songs eliciting mainly correct responses from the katydids varied considerably in overall structure and in the form of the cueing echeme (Fig. 6). The katydids accurately mimicked female cicada replies to simple songs containing only one type of echeme (e.g., Fig. 6A–C), songs with a non-cueing introductory section as well as separate cues (Fig. 6D–I), songs with more complex cueing sections (Fig. 6J, K), and even some species with extremely complex songs (Fig. 6L). The cueing echemes of these species ranged from simple isolated ticks to echemes of nearly two seconds' duration.

*C. leucoviridis* replied more erratically to very complex cicadettine songs (e.g., Fig. 6L–N), although even in these cases the correct song echemes often elicited a significantly greater fraction of the katydid replies than expected given the frequency of occurrence of cueing elements compared to other song echemes (see the rightmost column in Table 2). Only one cicada species tested, *Kikihia scutellaris* (Fig. 6M), consistently “fooled” the katydids.

*C. leucoviridis* became increasingly aroused or “primed” to respond while hearing cicada song or similar intense sound. For example, the katydids sometimes responded only to cueing elements at first, but later began replying to non-cueing elements as well. In playback trials, the first song phrases played were less likely than later phrases to elicit replies. Also, the katydids often temporarily responded to all sharp ambient sounds (coin clicks, keyboard taps, etc.) following episodes of loud, high-frequency sound (e.g., wind noise from car windows, crunching up plastic bags). Finally, *C. leucoviridis* demonstrated a reduced overall response to the songs of cicadettine species with lower-frequency (<<10 kHz) songs, for example *Pauropsalta melanopygia* and *Graminitigrina bolloni*.

### Demonstrations of predation following aggressive mimicry

Six of the trial demonstrations of *Chlorobalius leucoviridis* capturing and eating cicadas followed a similar pattern (e.g., Video S1, Video S2) that took only about two or three minutes: Soon after the cicada began to sing, one or more katydids began responding, with many clicks following male song cues. The male cicada then turned toward and began walking and/or flying towards a replying katydid while continuing to sing, just as we have observed in cicada pair-formation. Once the cicada came within reach, the katydid snared it with its fore- and often midlegs and subdued it by partially biting off the head, a behavior common to many predatory orthopterans [15 and pers. obs.]. In one case a katydid slowly moved towards the approaching cicada while clicking. Some trials ended with no response by either katydids or cicadas. Successful attraction of the cicada by the katydid was demonstrated for “pale grass cicada” (Fig. 6F), *Pauropsalta* “near walkeri”, and *Pauropsalta* sp. “Sandstone” (Fig. 6H). In addition, we have observed separately-caged cicadas of *Kobonga umbrimargo*, *K. apicans*, and *Pauropsalta* “near extrema” responding positively to *C. leucoviridis* clicks.

**Figure 7:**
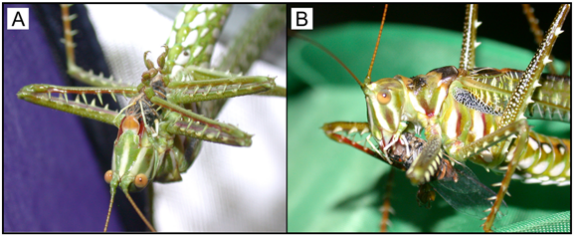
(A and B) *Chlorobalius leucoviridis* males devour cicadas that they have attracted with aggressive mimicry. In (A), note that the cicada is held between the tarsi with the tarsal claws held away from the prey and that the spines on the legs also do not contact the prey.

Captured prey were always held between the base of the tarsi, with the tarsal claws held away from the prey (Fig. 7A,B). In addition, the katydids often hung only by their hind legs to consume and sometimes to catch their prey (Fig. 7A). The formidable spines on the legs did not appear to come into contact with prey items [see also 15]. Captured cicadas were entirely consumed except for the forewings.

In most observations we noted that the replying *C. leucoviridis* bounced or jerked its body precisely in time with each click reply (Video S3). Tegminal movements made during male katydid song did not cause similar incidental body movements.

Individual *C. leucoviridis* demonstrated surprisingly different levels of overall responsiveness, and these “personalities” remained stable over many weeks of observations. All *C. leucoviridis* that were captured because they were found clicking in reply to cicada songs in the field were consistently responsive to cicada songs and other sharp noises. In contrast, of the males that were captured while singing, one was highly responsive while the other seven (and the two females) were much less so.

### Influence of light on *Chlorobalius* responses

Playbacks of model song cues in alternating treatments of light and darkness showed a strong effect of ambient light level on the katydid response. At least one of the two katydids responded to approximately half of the clicks in every light trial, while none replied to any clicks in any darkness trial (Fisher Exact 2-tailed *p* = 0.002). Male *C. leucoviridis* would often begin singing as soon as it became dark, and they seemed to switch from a “predatory” mode into a “courtship” mode with a decrease in light levels.

## Discussion

### Versatile acoustic mimicry in *Chlorobalius leucoviridis* and cicada song structure

Predatory *C. leucoviridis* katydids aggressively mimic Australian cicadas that use signal-response duets in sexual pair-formation (Tribe Cicadettini). The katydids respond to cues in male cicada songs with clicks from their tegminal stridulatory apparatus, mimicking the wing-flick sound made by conspecific female cicadas. Furthermore, the katydids respond with variable success to a variety of species-specific cicada songs. When a male cicada is drawn to within a few centimeters of a responding *C. leucoviridis*, the katydid deftly snares the cicada and eats it. While we have observed only one male cicada being attracted (but not captured) in the wild, the katydid phonoresponse is easily demonstrated through playbacks, and successful aggressive mimicry occurs readily in tent cages.

The versatility of *C. leucoviridis*' mimicry appears astonishing at first, given the complexity and species-specificity of Cicadettini songs. Especially striking is the ability of the katydid to produce correct responses to cicada songs that the predator species has never encountered (Fig. 6). However, in key respects Cicadettini songs are not that variable, and *C. leucoviridis'* versatility probably derives from the application of a few general rules. In most of the cicadettine species that we have observed, the female response is cued by a comparatively short song echeme that terminates abruptly, similar to the “trigger pulses” observed in many Orthopteran species with pair-forming duets [16]–[19]. In cicadettine cicadas and duetting Orthopterans, the species-specificity of the female's phonoresponse is likely explained by features of other song elements. *C. leucoviridis* presumably benefits from attracting male cicadettine cicadas of all available species, and therefore it has evolved a response that is not dependent on the form of the noncueing elements. Versatile aggressive mimicry of multiple *Photinus* prey species by *Photuris* fireflies has also been attributed to the general application of a single neural mechanism [20], [21].

### Predators and the evolution of acoustic sexual signals

The causes of song evolution are important in insect biology because the songs of singing insects are generally the most important trait affecting premating isolation [22]–[24] and because songs often diverge in diagnosable ways before other phenotypic attributes do (including genitalia). Several studies have demonstrated predators locating prey [25] and/or hosts [26] by their songs. Predator-prey arms races [27] have been proposed to account for important aspects of signaling behavior including call duration [20], [28], the timing and frequency of singing [29], and song structure [30],[31],[32 p. 97]. Such changes could facilitate speciation by changing allopatric populations in ways that isolate them upon re-establishment of sympatry [31], [33], [34].

Studies of behavioral evolution in cicadettine cicadas should take into account possible effects of persistent predation by aggressive mimics like *C. leucoviridis*. For example, even though *Kobonga oxleyi* (the species we observed being attracted by *C. leucoviridis*) has a structurally obvious song cue and an easily timed repetitive rhythm, we have found this species to be extremely resistant to our artificial signals. Poorly timed finger-snaps cause males of many species to become wary, with *K. oxleyi* an especially strong example. Perhaps persistent aggressive mimicry by *C. leucoviridis* has selected *K. oxleyi* males for greater sensitivity to the occasional poorly timed click. This possibility also suggests an additional evolutionary route for the cicada prey – the addition of “false cues” that elicit premature katydid replies without cueing female cicadas, whose response depends on a particular combination of song elements. Long-continued selection of this sort might account for the extraordinarily complex songs of many Australian cicadettine species (e.g., Fig. 6L, 6N) found in the arid, acacia-dominated habitats where *C. leucoviridis* is most common.

### Preadaptive origins of aggressive mimicry in *Chlorobalius*

Aggressive mimicry of the cicadettine acoustic duet requires a suite of complex traits including sound-generating structures, sound receptors, and neural processors capable of interpreting complex song patterns. These attributes are broadly present in most subfamilies of Tettigoniidae [35], including the Listroscelidinae [5], so it may not be surprising that an acoustic mimic of cicadettine cicadas has arisen from this family. Unfortunately, little else is known about the ecology of *C. leucoviridis* [5], so it is difficult to speculate whether the aggressive mimicry observed here had its origins in intraspecific acoustic behavior. So far, our observations suggest that *C. leucoviridis* does not use acoustic duets in pair-formation. We have not observed female katydids clicking to male katydid song (although females were observed clicking to cicadas), and the particular form of the male katydid song, a trill which lacks recognizable song cues, suggests a species in which females approach stationary males without replying.

The experimental finding that the *C. leucoviridis* click mechanism operates only during daylight is expected if the click response has evolved as a tool for capturing cicadas, and it is not expected if the behavior has evolved in the context of intraspecific communication. Cicada prey are all but strictly diurnal, while most *C. leucoviridis* male singing activity and flight occurs at night or at dusk [5 and pers. obs.]. We have never heard our caged *C. leucoviridis* responding to sharp ambient sounds at night, even though this frequently happens in daylight.

*C. leucoviridis* katydids often jerk or bounce their bodies in time with their click replies. This jerking movement is probably not necessary for the katydid to produce the click since they do not bounce while singing. Katydids of other types use leaf-shaking or tremulation combined with stridulation for intraspecific interactions [36], [37], including aggression. However, despite the fact that we have kept both male and female *C. leucoviridis* together in small (1.5 liter) cages for many weeks at a time, we have never observed the katydids acting aggressively toward one another. We have observed only rare instances of rapid clicking between individuals that seemed to be accidental cascading predatory click responses. A better explanation for the synchronized clicking and body-jerking behavior in *C. leucoviridis* is that the movement adds a visual component to the acoustic lure. Some male cicadas have been demonstrated to search visually as well as acoustically for female wing-flick responses [*Magicicada septendecim*, 14, and *Kikihia* sp. unpubl. data], and the behavior is probably widespread in the Cicadettini.

Future studies should provide a more detailed understanding of the origin of aggressive mimicry in *C. leucoviridis*. Adults are easily kept in captivity for weeks, and most individuals readily exhibit phonoresponses even in a highly artificial environment. Further phylogenetic insight into the origins of aggressive mimicry in this species will have to wait until more is known about the behavior and phylogenetic relationships of Tettigoniid katydids, which are currently under investigation [38]–[40].

## Methods

### Field collection and specimen storage

*Chlorobalius leucoviridis* adults were collected in the field during studies on Australian Cicadidae and kept in cages for up to five weeks, where they survived well on a diet of cicadas, small katydids and large flies, supplemented daily with sprayed water droplets. Cicadettine cicadas were collected opportunistically and caged 1–2 meters from caged *C. leucoviridis* while we traveled, and acoustic interactions between the cicadas and katydids were recorded opportunistically. The cages used were 1.5 liter mesh fabric “Port-a-Bug” cages obtained from Insect Lore, P.O. Box 1353, Shafter, CA.

### Song recording equipment and playback technique

Sound recordings were taken with a Marantz PMD-660 or PMD-670 digital flash recorder and a Sennheiser ME-62 omnidirectional microphone, sometimes mounted in a SONY PBR-330 parabolic reflector. Playbacks of cicada song to caged katydids were conducted using the speakers of a Macintosh G4 Powerbook computer, and the sounds were reproduced at intensities approximating natural cicada sound from a distance of 0.5–1 m. Some playback recordings were filtered to remove low-frequency background sounds. Environmental temperatures were measured with an Omega HH-25KF thermocouple (OMEGA Engineering, Stamford, CT). Acoustical analyses were conducted on a Macintosh G4 Powerbook using Raven version 1.3 software (Cornell Lab of Ornithology, Ithaca, NY). Time measurements (in ms) were taken from sonograms when possible, and rarely from filtered oscillograms. Measurements are accurate to approximately 1 ms.

When possible, the association of katydid reply clicks and cicada song cues was assessed with a one-tailed 2-by-2 binomial test. Song echemes, identified by separating gaps of ca. 60 ms or more, were classed as “cues” and “non-cues” based on our knowledge of song structure in each species. Katydid replies were classified as “correct” if they were placed in the gap following a song cue and “incorrect” if they occurred anywhere else in the song. The hypothesis of no association was rejected if the *p*-value was less than 0.05. If more than one katydid replied at the same time, the event was scored as a single reply. We considered only those interactions involving cicada species for which we have observed or tested the correct position of the female reply.

### Demonstrations of predation following aggressive mimicry

Demonstrations of predation were conducted on five different dates during 2005–2008 in an ordinary three-person camping tent, in each case using the katydids available to us at the time. All of the katydids were used in at least one trial. In each demonstration, one or more *Chlorobalius leucoviridis* katydids was released into the tent (where they typically rested on the upper surface) and allowed to remain undisturbed for several minutes. Then, one or more cicadas were introduced and any cicada-katydid interactions were observed for a maximum of fifteen minutes. Some interactions were video-recorded with a Fuji Finepix S9100 or Nikon Coolpix 995 digital camera. Cicadas used were *Pauropsalta* sp. “near walkeri”, *P. melanopygia*, *P*. sp. “Sandstone”, *Kobonga apicans*, *Urabunana marshalli*, *Cicadetta viridis*, and Undesc. genus, sp. “pale grass cicada” (see Table 2 and Fig. 6).

### Test of photosensitivity of katydid phonoresponse

Preliminary observations suggested that *Chlorobalius leucoviridis* katydids did not reply to cicada song in darkness, despite the fact that the katydids are active and sing at night. As a result, we tested the effect of light on *C. leucoviridis*' aggressive mimicry. In this experiment, two katydids were presented computer playbacks of 100 sharp click sounds at a uniform rate of two clicks per second, in alternating trials of ordinary incandescent room light and near-complete darkness (six light trials, six dark trials). Each trial was preceded by approximately six minutes of light- or dark-acclimation, and the ambient temperature was 24.5°C. In the latter three darkness trials the number of playback clicks was increased to 200 to check for a more delayed response. (The experiment was initially conducted by hand using coin clicks, with the same outcome.)

## Supporting Information

Video S1 Digital camera A/V footage of a male cicada (Undesc. genus, sp. “pale grass cicada”) being attracted and captured by a clicking *Chlorobalius leucoviridis* male. The camera was capable of recording the *C. leucoviridis* clicks (faint) but not the high-frequency cicada sound.
(17.28 MB MOV)

Video S2 Digital camera video footage of a male cicada (Undesc. genus, sp. “pale grass cicada”) being attracted and captured by a clicking *Chlorobalius leucoviridis* male. The male cicada's abdomen moves up and down in time with his song phrases. The resolution is not fine enough to observe movements of the katydid's tegmina. No sound is available.
(3.05 MB MOV)

Video S3 Digital-camera A/V footage of a *Chlorobalius leucoviridis* clicking in response to the song of a male cicada (Undesc. genus, sp. “pale grass cicada”), showing how the katydid bounces its body in time with its click sounds. The cicada's song frequencies are too high for the video camera, but the katydid clicks are clearly audible.
(15.97 MB MOV)

Audio S1 Recording of *Kobonga oxleyi* cicada song with reply clicks from a *Chlorobalius leucoviridis* katydid. A section of this recording is illustrated in Fig. 5.
(0.64 MB MP3)

Audio S2 Recording of *Pauropsalta* sp. “Sandstone” song with reply clicks from a *Chlorobalius leucoviridis* katydid. A section of this recording is illustrated in Fig. 6H.
(0.80 MB MP3)
